# Cost-effectiveness of 12 months of capecitabine as adjuvant chemotherapy for stage III colon cancer: preplanned cost-effectiveness analysis of the JFMC37-0801 study

**DOI:** 10.1007/s10198-021-01418-6

**Published:** 2022-01-24

**Authors:** Hidetoshi Shibahara, Takeru Shiroiwa, Megumi Ishiguro, Masato Nakamura, Junichi Hasegawa, Shigeki Yamaguchi, Yuriko Masuda, Junichi Sakamoto, Naohiro Tomita, Takashi Fukuda

**Affiliations:** 1 CRECON Medical Assessment Inc., Tokyo, Japan; 2 Department of Medical Service Management, Graduate School of Medical Service Management, International University of Health and Welfare, Tokyo, Japan; 3 Center for Outcomes Research and Economic Evaluation for Health, National Institute of Public Health (NIPH), Wako, Japan; 4 Department of Chemotherapy and Oncosurgery, Tokyo Medical and Dental University Medical Hospital, Tokyo, Japan; 5 Aizawa Comprehensive Cancer Center, Aizawa Hospital, Matsumoto, Japan; 6 Department of Surgery, Osaka Rosai Hospital, Osaka, Japan; 7 Department of Colorectal Surgery, Saitama Medical University International Medical Center, Saitama, Japan; 8 Tokai Central Hospital, Kakamigahara, Japan; 9 Epidemiological and Clinical Research Information Network, Kyoto, Japan; 10 Cancer Treatment Center, Toyonaka Municipal Hospital, Toyonaka, Japan; 11 Department of Surgery, Hyogo College of Medicine, Nishinomiya, Japan; 12 Center for Outcomes Research and Economic Evaluation for Health, National Institute of Public Health, Saitama, Japan

**Keywords:** Cost-effectiveness, Capecitabine, Adjuvant chemotherapy, Colon cancer, JFMC37-0801 study, I19

## Abstract

**Objectives:**

We evaluated the cost-effectiveness of a 12-month regimen of oral capecitabine versus a standard 6-month regimen as postoperative adjuvant chemotherapy for stage III colon cancer.

**Methods:**

We utilized patient-level data from a multi-institutional randomized controlled trial (JFMC37-0801) that investigated prolonged oral fluoropyrimidine monotherapy. The analysis considered three health states: stable disease, post-metastasis, and death. A parametric statistical model with a cure model was used to estimate the survival curve. The analysis was conducted from the Japanese public healthcare payer’s perspective, considering only direct medical costs. A lifetime horizon was used, with a discount rate of 2% for both cost and health outcomes. Health outcomes were evaluated in terms of quality-adjusted life-years (QALYs).

**Results:**

The estimated cure rates for colon cancer were 0.726 [95% confidence interval (CI) 0.676–0.776] and 0.694 (95% CI 0.655–0.733) with the 12- and 6-month regimens, respectively; and the estimated 5-year relapse-free survival rates were 74.4% and 69.8%, respectively. The estimated lifetime cost for 12 months of capecitabine was JPY 3.365 million (USD 31,159), compared with JPY 3.376 million (USD 31,262) for 6 months. The estimated QALY were 12.48 and 11.77 for the 12- and 6-month regimens, respectively. Thus, the 12-month capecitabine regimen was dominant. Using a willingness-to-pay threshold of JPY 5 million per QALY, we determined a 97.4% probability that the 12-month capecitabine regimen is more cost-effective than the 6-month regimen.

**Conclusions:**

Twelve months of capecitabine is the favorable option for postoperative adjuvant chemotherapy for stage III colon cancer from the perspective of cost-effectiveness.

**Supplementary Information:**

The online version contains supplementary material available at 10.1007/s10198-021-01418-6.

## Key points for decision-makers

**Table:** 

Although the JFMC37-0801 study suggested that a 12-month regimen of oral capecitabine improves survival compared with a 6-month regimen for patients with stage III colorectal cancer (CRC), there are concerns about increased adverse events, impaired quality of life, and higher medication costs due to the extended treatment period.
This study evaluated the cost-effectiveness of a 12-month regimen with that of a 6-month regimen, and the 12-month capecitabine regimen was dominant.
CAPOX (capecitabine plus oxaliplatin) is widely accepted as the international standard postoperative adjuvant chemotherapy for stage III CRC. On the other hand, oxaliplatin has been rejected by some patients due to its neurotoxicity. Oral fluoropyrimidine monotherapy is the preferred adjuvant therapy in Japan, and our study results suggest that 12 months of capecitabine monotherapy may be a potential adjuvant treatment alternative, especially for neurotoxicity-averse patients.

## Introduction

Colorectal cancer (CRC) is among the commonest malignant neoplasms worldwide. In Japan, the incidence of CRC has increased lately, with approximately 152,100 new CRC cases reported in 2018 [1].

Postoperative adjuvant chemotherapy for patients with stage III CRC is the internationally accepted standard of care for improving patient survival [2, 3]. Based on the results of three randomized controlled trials (RCTs) conducted in Europe and the United States, 6-month oxaliplatin-containing regimens and the combination of capecitabine and oxaliplatin (CAPOX) are widely accepted as the standard adjuvant chemotherapy regimens for stage III CRC [4–6]. On the other hand, treatment with oxaliplatin-containing regimens often causes neurotoxicity, making it difficult to continue treatment. Oral fluoropyrimidine monotherapy—such as capecitabine, tegafur-uracil and leucovorin (UFT/LV), and tegafur with gimeracil and oteracil (S-1)—is a viable option, especially for older patients and patients who have rejected the neurotoxicity of oxaliplatin. In Japan, oral fluoropyrimidine monotherapy is preferred in the adjuvant setting, with demonstrated favorable outcomes in Japanese RCTs [7–10]. In these studies, 6-month regimens were investigated as the standard following the treatment duration in clinical trials of oxaliplatin-containing regimens in Europe and the United States. However, whether longer treatment can improve prognosis has not been fully investigated.

To investigate the hypothesized superiority of prolonged oral fluoropyrimidine monotherapy, the JFMC37-0801 study (UMIN-CTR; UMIN000001367) [11], a multi-institutional RCT, was launched in September 2009, comparing 12-month capecitabine with the standard 6-month regimen as postoperative adjuvant chemotherapy for stage III colon cancer. The primary endpoint was disease-free survival (DFS), and the secondary endpoints were relapse-free survival (RFS) and overall survival (OS). A total of 1304 patients were randomized (12 M group, *n* = 650; 6 M group, *n* = 654). The 3- and 5-year DFS rates were 75.3% and 68.7%, respectively, in the study group (12 M group) and 70.0% and 65.3%, respectively, in the control group (6 M group) [hazard ratio (HR) 0.858, 90% confidence interval (CI): 0.732–1.004, *p* = 0.0549]. The 5-year RFS rates were 74.1% and 69.3% in the 12 M and 6 M groups, respectively (HR 0.796, 90% CI 0.670–0.945, *p* = 0.0143), and the 5-year OS rates were 87.6% and 83.2%, respectively (HR 0.727, 90% CI 0.575–0.919, p = 0.0124). In other words, in terms of DFS, superiority of the 12-month regimen was not demonstrated, but OS and RFS were significantly longer in association with the 12-month regimen. The incidence of overall grade 3–4 adverse events (AEs) was comparable between the groups, while the 12 M group had a higher cumulative incidence of hand-foot syndrome (HFS) compared with the 6 M group (23 versus 17%, *p* = 0.011) [12]. Based on these results, 12-month capecitabine monotherapy may be an option for adjuvant treatment, especially for patients particularly prone or averse to neurotoxicity.

Although the 12-month capecitabine regimen may have some survival benefit, there are concerns about an increased risk of associated AEs, including HFS, with impaired quality of life (QOL) and higher medication costs due to the extended treatment period. Focusing on the latter issue, the JFMC37-0801 study group planned a cost-effectiveness analysis of the 12-month capecitabine regimen compared with the standard 6-month regimen. Here, we report the results of this additional analysis using prospectively collected treatment, survival, QOL, and medical resource utilization data.

## Methods

### Patients and treatment regimens

We analyzed patient-level data from a multi-institutional, open-label RCT (JFMC37-0801) [11] wherein patients aged 20–80 years with curatively resected stage III (T1–4, N1–2, M0) colon cancer were randomly assigned to either 12 or 6 months of capecitabine. Capecitabine (1250 mg/m^2^) was orally administered twice daily after meals for 14 consecutive days, followed by 7 days of rest; this 3-week period comprised one course. The study group (12 M group) and the control group (6 M group) received 16 and 8 courses, respectively. The assigned treatment was started within 8 postoperative weeks. After completing the treatment protocol, patients were followed up according to a predefined surveillance schedule. The study was conducted in accordance with the Declaration of Helsinki and the Ethical Guidelines for Clinical Research in Japan; it was approved by the institutional review boards of each participating institutions. Written informed consent was received from all patients before their enrollment. Additional details, including dose modifications, were provided previously [12].

### Framework of the economic analysis

The cost-effectiveness analysis of the 12-month capecitabine compared with the 6-month regimens was conducted in accordance with the guidelines on cost-effectiveness evaluation disseminated by Japan’s Ministry of Health, Labour and Welfare (MHLW) [13]. The health outcomes of each intervention were evaluated in terms of quality-adjusted life-years (QALYs). The analysis was conducted from the Japanese public healthcare payer’s perspective and included only direct medical costs. A lifetime horizon was used, and the discount rate was set at 2% for both costs and health outcomes. The cost-effectiveness was determined by the incremental cost-effectiveness ratio (ICER), and the ICER reference value to be judged as cost-effective was determined to be JPY 5 million (USD 46,296) per QALY, referring to the willingness-to-pay threshold in Japan [14–16]. Unit costs were based on the 2018 Japanese fee schedule and drug tariffs, each defined by the MHLW at an exchange rate of USD 1 = JPY 108 (December 2019), as reported by the Bank of Japan [17]. Analyses were performed using SAS^®^ 9.4 (SAS Institute Inc., Cary, NC, USA.) and Microsoft Excel® for Office 365 (Microsoft Corp., Redmond, WA, USA).

### Survival analysis

The analysis was conducted using a partitioned survival analysis, comprising area under the curve interpretation from a set of mutually exclusive survival curves to determine state membership, exploiting the unidirectional nature of transitions in a progressive model [18]. The analysis model considered three health states: stable disease, post-metastasis, and death. Stable disease was defined as without metastasis or death from any cause. In the JFMC37-0801 study, RFS was defined as survival from randomization without recurrence or death from any cause. Thus, OS can be divided into pre-metastasis and post-metastasis with consideration of RFS and OS.

Although some colon cancer patients develop metastases, some cancers can be completely cured. The population was thus a mixture of two subpopulations of cured patients and uncured patients, and RFS and OS survival were expected to differ between the two subpopulations. RFS and OS must be estimated with the assumption that they are different for each subpopulation; however, standard survival models do not assume two different populations. The cure model enables covariates to have different influences on cured patients and uncured patients [19]. The cure model approach was applied to estimate the RFS and OS survival curves. The survival function can be shown in the following way:

*S* (*t*) = *p* + (1 − *p*) *S*(*t*) (where *p* is the probability of cure).

The cure rate was estimated by the RFS data from the JFMC37-0801 study. The definitions of OS and RFS in the JFMC37-0801 study included other causes of death. Immediately after the start of the study, the proportion of cancer-related events such as cancer recurrence and cancer-related death is expected to be high, while the proportion of other causes of death is expected to increase over time. OS and RFS in non-cured patients were estimated separately for “cancer-related events” and “other causes of death” because the breakdown of events defined as OS and RFS in non-cured was expected to change over time. The survival function for non-cured patients was investigated in four distributions: Weibull, log-logistic, log-normal, and exponential. The suitability of the fitted models was assessed by a combination of statistical fit, clinical plausibility, and visual examination of the parametric model curves [20]. Statistical goodness-of-fit was assessed based on the Akaike information criterion (AIC) and Bayesian information criterion (BIC), with smaller values indicating a better fit. For the statistically well-fit models, the clinical plausibility of the slope and the curve tails were assessed. To account for increased mortality with aging, the risk of other causes of death after 60 months was assumed to be equivalent to that of the general population of the same age, and a nonparametric survival function with life tables for the general population was applied. For cancer-related deaths, the estimated parametric curve was applied throughout the lifetime. The mortality in cured patients was given as a nonparametric mortality using life tables for the general population.

### QALY estimation

The expected QALY obtained for each treatment was calculated as the survival proportion for each health state multiplied by the corresponding utility value. The utility value for stable disease was taken from an auxiliary study to the JFMC37-0801 study [21]. Patients who agreed to participate in a health-related QOL (HRQOL) survey were evaluated using the EuroQOL 5 dimensions 3-level (EQ-5D-3L) tool at the start of the protocol treatment, and after 3, 6, 9, 12, 15, 18, 24, 36, 48, and 60 months. When a patient relapsed, the patient was excluded for ethical reasons. The EQ-5D-3L scores were converted to utility values using Japanese tariffs [22]. After we calculated basic statistics, the utility values were applied to a linear mixed model of repeated measures. Utility values were estimated for each treatment group up to 12 months because HRQOL might vary between the treatment groups due to divergent frequencies and severity of AEs.

After 12 months, the estimation was performed without distinguishing between treatment groups. The utility values were adjusted with respect to baseline score, treatment, time, and treatment-by-time interaction during the treatment period, and they were only adjusted with respect to baseline score and time after 12 months. The random effects of individual patient factors were included in the model. Relapse and death were censored, and other missing values were supplemented by the last observation carried forward method. After the study period (60 months), the utility value of stable disease was set using values from the general population [23] because the estimated utility at 60 months was higher than that of the general population, and patients with a recurrence-free period greater than 5 years were assumed comparable to the general population.

Since the QOL data were not captured after patients relapsed, the utility value for the post-metastasis period, set at 0.705, was taken from a published report by Huxley et al. [24] through a targeted literature review.

### Medical resource utilization

The cost parameters were based on patient-level data from the JFMC37-0801 study [11] and its auxiliary study. Capecitabine costs were calculated using the actual dose data recorded in the JFMC37-0801 study’s case report form. The costs of stable disease and post-metastasis were calculated using the claims data obtained from the patients who agreed to participate in the HRQOL survey. The medical fee reimbursement claimed by medical institutions was selected as each state cost, which included the monthly total billed amounts for all inpatient and outpatient medical interventions [visit costs, tests, treatments, including drugs (excluding capecitabine) and treatment for AEs].

The annual costs of stable disease were calculated separately by treatment group from baseline to 2 years. The total cost of months 1–12 for each treatment group was defined by the cost of stable disease in the first cycle, and months 13–24 were used to define the cost of stable disease in the second cycle. Thereafter, both groups were tabulated together because the observational costs during the stable disease period were assumed to be equal in both groups. The costs of stable disease were not considered after 5 years, as Japanese guidelines recommended a monitoring period of 5 postoperative years [2]. The expected cost of the stable disease obtained from each treatment group was calculated as the survival proportion in the RFS state multiplied by the costs of stable disease.

The costs of the post-metastasis period were calculated using data from relapsed patients tabulated on a monthly basis. There was a trend toward higher costs immediately after recurrence and lower costs thereafter, indicating that annual costs are not constant. Given that the model was a partitioned survival model, it was not possible to change the cost for patients with recurrence from year to year. The monthly cost was multiplied by the survival proportion in each month for relapse (up to 60 months), and the event cost for relapse cases was set (undiscounted).

### Sensitivity analysis

Uncertainty over the post-metastasis costs were assessed using one-way sensitivity analysis (JPY 1–8 million) because it could only be calculated from 25 patients, and the discount rate was not considered in the analytical model, resulting in high uncertainty. In addition, since the post-metastasis utility value was not available in the JFMC37-0801 study, and value reported overseas by Huxley et al. was used, a one-way sensitivity analysis was also performed for the post-metastasis utility value (0.600–0.800). Since data on the variability of the values were not available, a wide range of assumed values was used in the analysis. Furthermore, to assess the impact of the distribution selection of the survival function, a scenario analysis was conducted in which different distributions were selected.

The bootstrap method (10,000 resamples) was used for the probabilistic sensitivity analysis, and a cost-effectiveness acceptability curve was created [25]. In the bootstrap method, all parameters obtained from JFMC37-0801 were resampled and subjected to sensitivity analysis, except for the utility for disease progression taken from the literature. The utility for disease progression was randomly sampled by probability distribution following a beta distribution.

## Results

### Survival curve estimation

A total of 1304 patients (650 in the 12 M group, 654 in the 6 M group), randomized in the JFMC37-0801 study, were analyzed. The mean age of the patients was 63.9 ± 9.2 years, and 46.7% were female. The patients’ baseline clinical characteristics are summarized in the supplementary material.

**Fig. 1:**
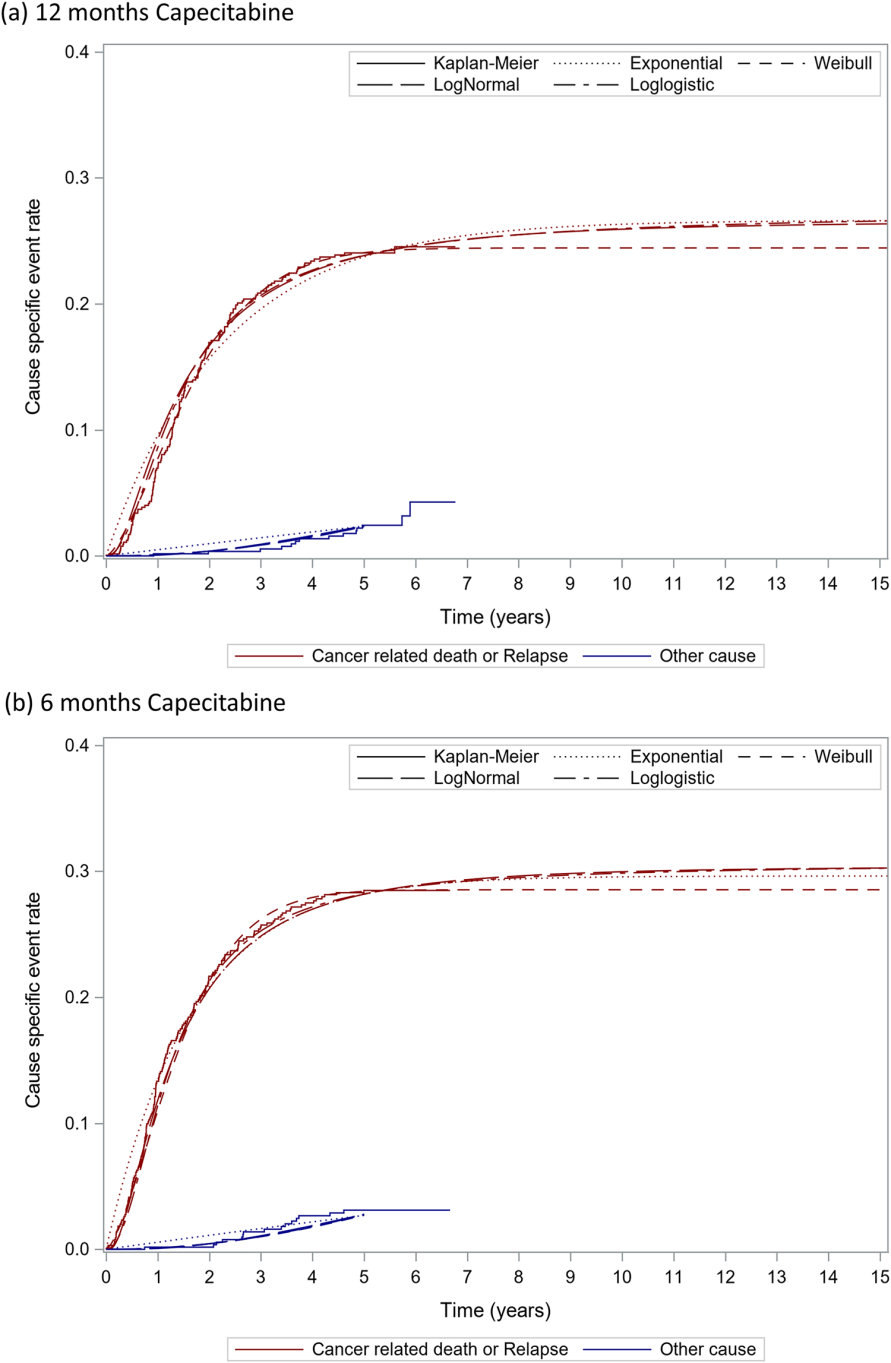
Estimated cause-specific RFS. RFS, relapse-free survival. The AICs of cancer related events for the exponential, Weibull, lognormal and log-logistic distributions were 2427.8, 2373.8, 2391.4 and 2374.7, respectively and BICs were 2438.1, 2389.4, 2406.9 and 2390.2, respectively. The AICs of other cause death for the exponential, Weibull, lognormal and log-logistic distributions were 368.0, 365.5, 355.7 and 356.3, respectively and BICs were 378.4, 372.0, 371.3 and 371.8, respectively

**Fig. 2:**
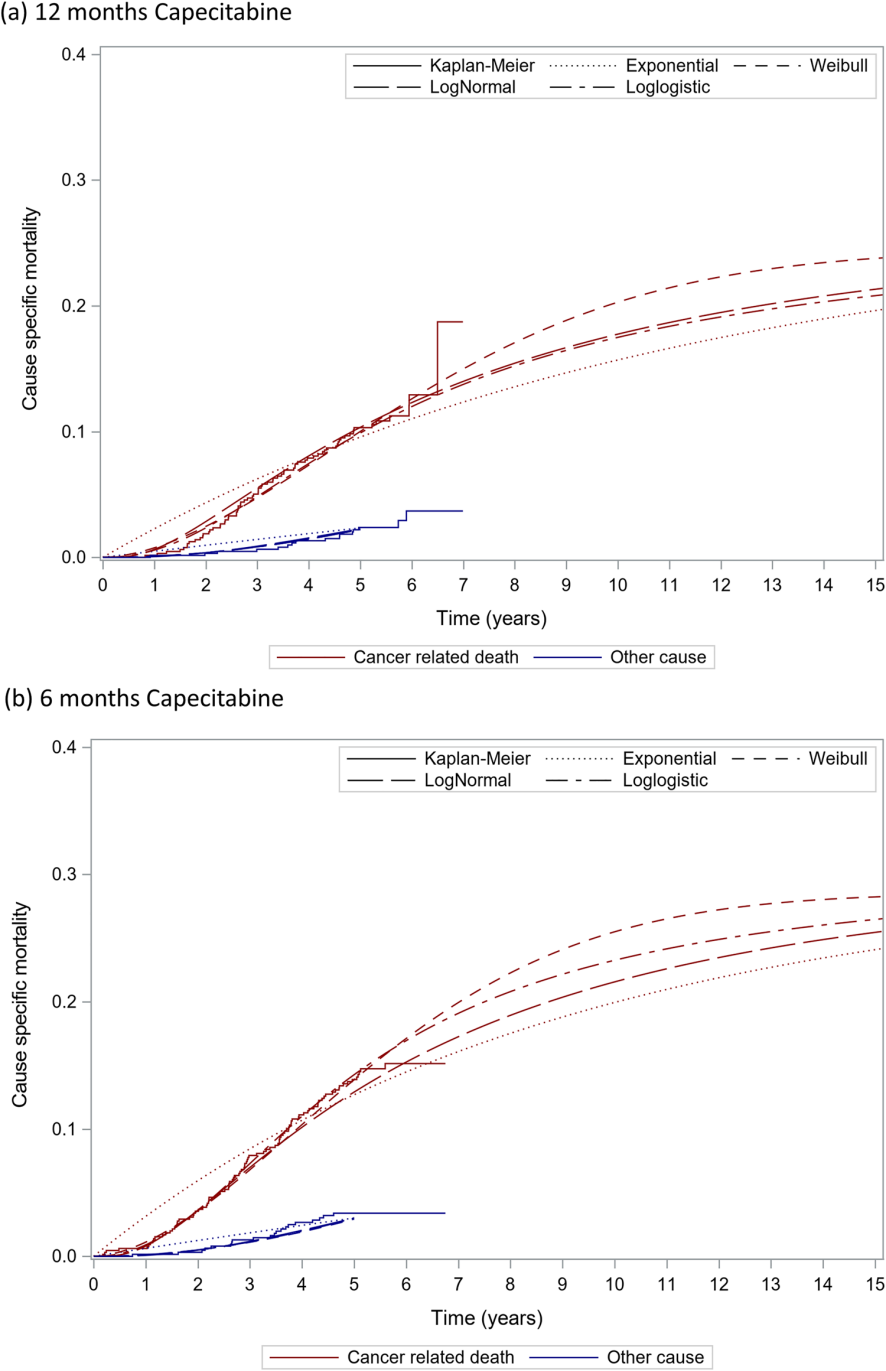
Estimated cause-specific OS. OS, overall survival. The AICs of cancer related death for the exponential, Weibull, lognormal and log-logistic distributions were 1553.8, 1508.5, 1511.3 and 1503.4, respectively and BICs were 1564.1, 1524.0, 1526.8 and 1518.9, respectively. The AICs of other cause death for the exponential, Weibull, lognormal and log-logistic distributions were 451.4, 438.8, 437.6 and 438.5, respectively and BICs were 461.7, 454.4, 453.1 and 454.0, respectively

The estimated cure rates for colon cancer were 0.726 (95% CI 0.676–0.776) and 0.694 (95% CI 0.655–0.733) for the 12- and 6-month capecitabine regimens, respectively. Figure 1 displays the estimated cause-specific RFS curves for non-cured patients. In the survival function of cancer-related death or relapse, the AIC and BIC values were lower for the Weibull (AIC: 2373.8, BIC: 2389.4) and log-logistic distributions (AIC: 2374.7, BIC: 2390.2) than for the other distributions. Visual examination revealed that the Weibull distribution had a slightly lower event risk over time than the other distributions. Although the recurrence risk for colon cancer after 5 years is known to be low, the Weibull distribution was considered to be even lower than that. The log-logistic curve was used in the survival function of cancer-related events in non-cured patients as the most reasonable fit. Figure 2 displays the estimated cause-specific OS curves for non-cured patients. In the survival function of cancer-related death, the logistic distribution had the smallest AIC and BIC and was visually validated. The log-logistic curve was used in the survival function of cancer-related death in non-cured patients. For other causes of death, the log-logistic distribution was used, which was the best statistical fit.

**Fig. 3:**
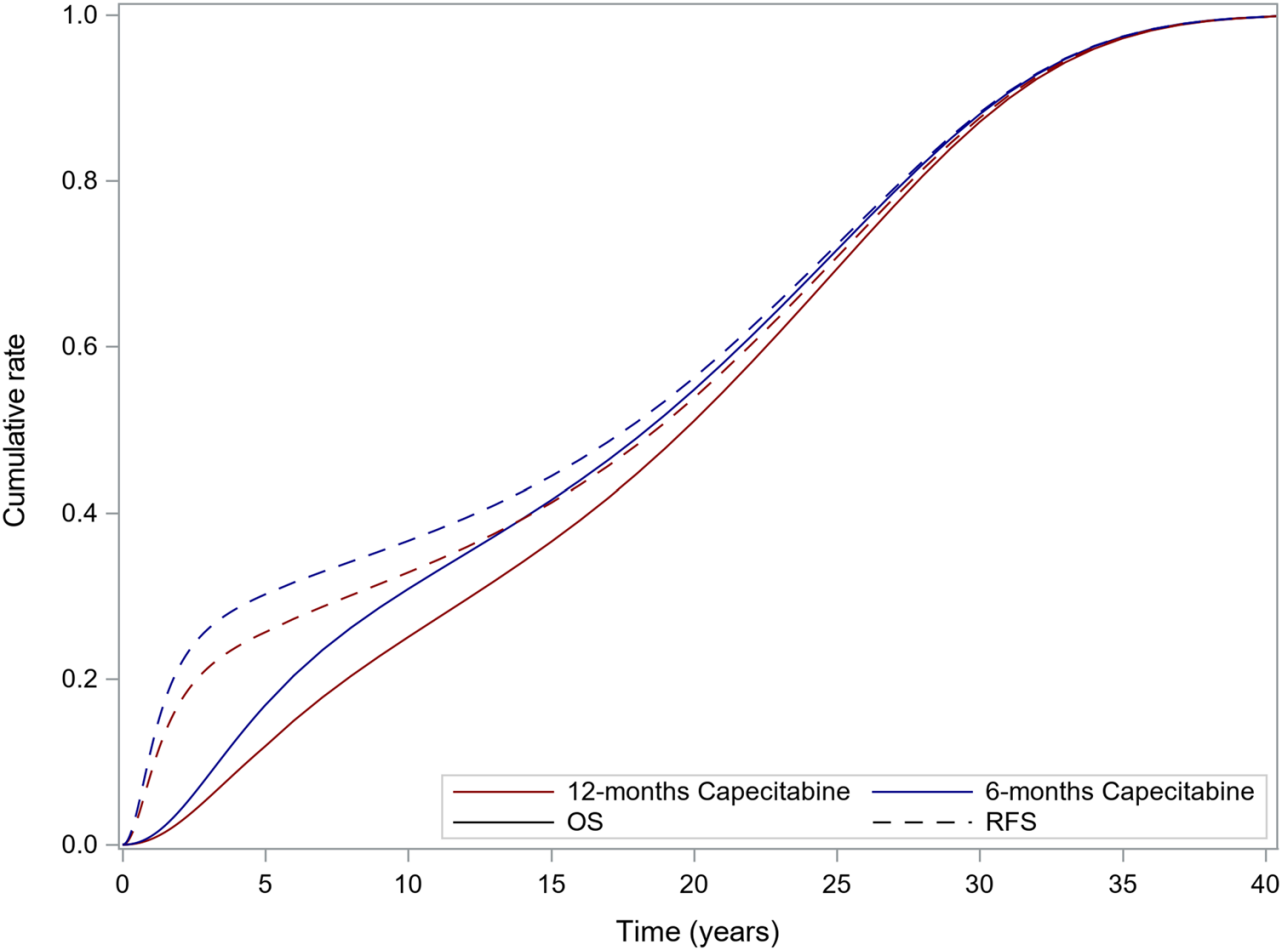
Integrated OS and RFS of the whole population. OS, overall survival; RFS, relapse-free survival

Figure 3 displays the integrated OS and RFS of the whole population estimated by the cure model. The estimated 5-year RFS rates were 0.744 and 0.698 with the 12- and 6-month capecitabine regimens, respectively, and the estimated 5-year of OS rates were 0.881 and 0.832, respectively. The mean OS durations were 18.6 and 17.5 years with 12 and 6 months of capecitabine treatment, respectively (no discount).

### Utilities

Among the 171 patients (90 in the 12 M group, 81 in the 6 M group) enrolled in the HRQOL survey, data were available and analyzed for 166 (87 in the 12 M group, 79 in the 6 M group). The patients’ baseline clinical characteristics, the CONSORT diagram of the QOL survey participants, and the collection rates of the EQ-5D-3L survey at each time point are shown in the supplementary material. The collection rates in the 12 M group and 6 M groups were 80.5% and 83.6%, respectively, at 12 months, and 64.0% and 71.7%, respectively, at 60 months. The estimated utility scores at 6 and 12 months were 0.861 and 0.901 in the 12 M group and 0.847 and 0.890 in the 6 M group. Although the utility values were thought to be different due to the differences in treatment duration and AE incidence, the analysis showed that the utility scores during the treatment period were similar between the treatment groups. Table 1 summarizes the utilities used in the cost-effectiveness analysis.

**Table 1:** Estimated results of EQ-5D scores and the model parameters of utilities

Period	12 months (SE)	6 months (SE)	References
*Stable disease*			
Month 1	0.853 (0.0162)	0.842 (0.0152)	Additional study of JFMC37-0801 [21]
Month 3	0.854 (0.0162)	0.860 (0.0152)	
Month 6	0.861 (0.0163)	0.847 (0.0152)	
Month 9	0.900 (0.0170)	0.874 (0.0153)	
Month 12	0.901 (0.0175)	0.890 (0.0154)	
Month 15	0.915 (0.0123)	0.915 (0.0123)	
Month 18	0.917 (0.0124)	0.917 (0.0124)	
Month 24	0.918 (0.0124)	0.918 (0.0124)	
Month 36	0.907 (0.0125)	0.907 (0.0125)	
Month 48	0.904 (0.0126)	0.904 (0.0126)	
Month 60	0.913 (0.0127)	0.913 (0.0127)	
Thereafter	0.847	0.847	Shiroiwa et al. [23]
*Post-metastasis*	0.705	0.705	Huxley et al. [24]

### Medical resource utilization

Capecitabine dose data were collected for 1278 patients (636 in the 12 M group, 642 in the 6 M group) who received capecitabine at least once in the JFMC37-0801 study. The actual dose during the study period averaged 1786 tablets in the 12 M group and 1168 tablets in the 6 M group (1 tablet contains 300 mg of capecitabine). The cost of capecitabine during the treatment period was JPY 639,327 (USD 5920) per patient in the 12 M group and JPY 418,298 (USD 3873) per patient in the 6 M group.

The state costs were calculated from the claim data for 108 patients (61 in the 12 M group, 47 in the 6 M group). The patients’ baseline clinical characteristics, claims data collection rate, and the CONSORT diagram of the cost population are shown in the supplementary material. The collection rates decreased with time during the study period. The costs of stable disease for 1–12 months and 13–24 months were JPY 1,105,107 (USD 10,232) and JPY 237,066 (USD 2195), respectively, in the 12 M group, and JPY 817,143 (USD 7566) and JPY 227,872 (USD 2110), respectively, in the 6 M group. The cost of post-metastasis treatment was calculated based on 25 cases at JPY 7,631,672 (USD 70,664) per case. The monthly cost and survival probability for each month with metastasis are shown in the supplementary material. Table 2 summarizes the cost parameters.

**Table 2:** Model parameters of costs

	12 months	6 months
*Drug cost of capecitabine (during treatment period)*		
Dose (tablets)	1786	1168
Cost/tablet (JYP)^a^	358	358
Drug cost (JYP)	639,327	418,298
State costs		
*Stable disease (JYP/year)*		
1–12 months	1,105,107	817,143
13–24 months	237,066	227,872
25–36 months	175,281	175,281
37–48 months	120,152	120,152
49–60 months	120,152	120,152
Thereafter	0	0
Post-metastasis^b^ (JPY/case)	7,631,672	7,631,672

*JPY* Japanese yen

^a^Xeloda^®^ national health insurance (NHI) drug price for 2018. NHI drug price is price to be reimbursed to hospitals and pharmacies (“health-insurance medical service providers”) under the national health insurance programs

^b^The post-metastatic costs were not considered after 5 years. From the start of treatment to 3 years, the annual cost was calculated using the data for each year, and for the 4th and 5th years, the data for 2 years were combined to calculate the annual cost

### Cost-effectiveness analysis

Table 3 displays the results of the cost-effectiveness analysis. Although 12 months of capecitabine therapy was more costly than 6 months of capecitabine in the stable disease state [JPY 1.545 million (USD 14,308) versus JPY 1.229 million (USD 11,381)], the cost of post-metastasis was lower for the 12 M group [JPY 1.819 million (USD 16,851) versus JPY 2.147 million (USD 19,891)]. The total expected medical costs of 12 months of capecitabine were JPY 3.365 million (USD 31,159)—a savings of JPY 11,146 (USD 103) compared with 6 months of capecitabine [JPY 3.376 million (USD 31,262)]. In terms of health outcomes, the 12-month capecitabine regimen was associated with higher QALYs from baseline than the 6-month regimen in the stable disease state (11.44 QALYs versus 10.85 QALYs) and the post-metastasis state (1.04 QALYs vs. 0.92 QALYs) due to prolonged RFS and OS. The 12-month capecitabine regimen was associated with an overall mean of 12.48 QALYs compared with 11.77 QALYs for the 6-month regimen, for a difference of 0.71 QALYs. Thus, the 12-month capecitabine regimen was dominant to the 6-month capecitabine regimen.

**Table 3:** Results of cost-effectiveness analysis

Variable	12 months	6 months	Difference
Total cost (JPY)	3,365,120	3,376,266	− 11,146
Stable disease (JPY)	1,545,260	1,229,129	316,131
Post-metastasis (JPY)	1,819,860	2,147,137	− 327,277
Total QALY gain (QALY)	12.481	11.774	0.707
Stable disease (QALY)	11.440	10.853	0.587
Post-metastasis (QALY)	1.041	0.921	0.120
ICER	Dominant	–	–

*JPY* Japanese yen, *QALY* quality-adjusted life-years, *ICER* incremental cost-effectiveness ratio

In the one-way sensitivity analysis, the ICER increased with lower post-metastasis costs. At JPY 1 million per event, the ICER increased to JPY 0.387 million (USD 3581) per QALY. When the metastasis cost was lower than JPY 7.376 million per event, the total medical cost for 12 months of capecitabine became higher than that for 6 months of capecitabine. However, the 12-month regimen remained dominant or highly cost-effective when the metastasis costs were between JPY 1 million and JPY 8 million. When the utility for the post-metastasis changed between 0.600 and 0.800, the incremental QALYs associated with the 12-month regimen was 0.689–0.723, and the 12-month capecitabine regimen remained dominant (Table 4). Changes in the utility of post-metastasis only had a minor impact on the analysis results.

**Table 4:** Results of scenario analysis

Group	Cost (JPY)	Incremental cost (JPY)	Effectiveness (QALY)	Incremental effectiveness (QALY)	ICER (JPY/QALY)
*One-way sensitivity analysis (metastasis cost: JPY 1 million)*					
12 months	1,783,722	273,247	12.481	0.707	386,697
6 months	1,510,475	–	11.774	–	–
*One-way sensitivity analysis (metastasis cost: JPY 8 million)*					
12 months	3,452,953	− 26,941	12.481	0.707	Dominant
6 months	3,479,894	–	11.774	–	–
*One-way sensitivity analysis (metastasis utility: 0.6)*					
12 months	3,365,120	− 11,146	12.326	0.689	Dominant
6 months	3,376,266	–	11.637	–	–
*One-way sensitivity analysis (metastasis utility: 0.8)*					
12 months	3,365,120	− 11,146	12.621	0.723	Dominant
6 months	3,376,266	–	11.898	–	–
*Scenario analysis (using the Weibull distribution for the RFS survival function for cancer-related events)*					
12 months	3,367,200	− 4997	12.513	0.718	Dominant
6 months	3,372,197	–	11.795	–	–

*JPY* Japanese yen, *QALY* quality-adjusted life-years, *ICER* incremental cost-effectiveness ratio

In the selection of the distribution of the RFS survival function for cancer-related events, a scenario analysis was performed using the Weibull distribution because the AIC and BIC of the logistic and Weibull distributions were similar. The results of the scenario analysis showed that the costs and QALYs were similar to the base-case analysis, and the 12-month capecitabine regimen remained dominant (Table 4).

**Fig. 4:**
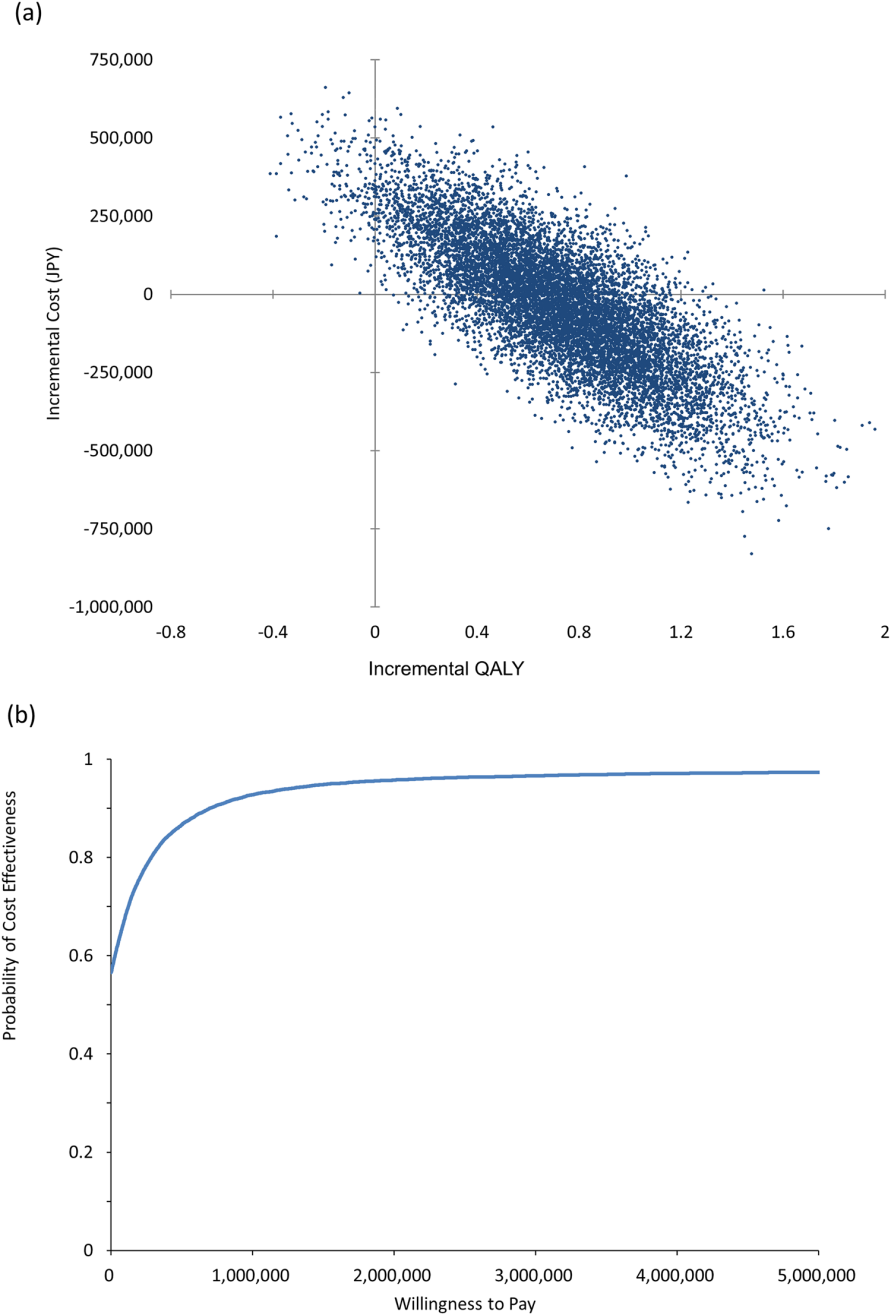
Result of probabilistic sensitivity analysis. QALY, quality-adjusted life year. **a** Scatter plot of incremental effectiveness and incremental cost; **b** acceptability curve

Figure 4 presents the cost-effectiveness acceptability curve and the scatter plot according to probabilistic sensitivity analysis. The 12-month capecitabine regimen was dominant, with a probability of 56.6%, and there was a 97.4% probability that the ICER of the 12-month regimen relative to the 6-month regimen was below JPY 5 million per QALY.

## Discussion

The present study evaluated the cost-effectiveness of 12 months of capecitabine as postoperative adjuvant chemotherapy for stage III colon cancer compared with a standard 6-month regimen from the Japanese public healthcare payer’s perspective. In the base-case analysis, the 12-month regimen was less expensive and was associated with more QALYs than the 6-month regimen, and thus was considered dominant throughout the lifetime. Sensitivity analyses support the dominance of the 12-month capecitabine regimen. The bootstrap method was used for the probabilistic sensitivity analysis because the Monte Carlo simulation method requires the distribution of each parameter, and it is difficult to consider all of their correlations, while the bootstrap method is nonparametric and, therefore, does not require such information. Given a willingness-to-pay threshold of JPY 5 million per QALY, the probability of the 12-month capecitabine regimen being more cost-effective than the 6-month regimen was 97.4%.

Based on these cost-effectiveness analysis results and the OS and RFS extension confirmed in the JFMC37-0801 study, the 12-month capecitabine regimen was an acceptable option for postoperative adjuvant chemotherapy for stage III colon cancer.

In this analysis, a parametric statistical model was employed to estimate the survival curve. Standard parametric distributions (curves shown in the supplementary material) may not satisfactorily fit the complex survival function; therefore, alternative approaches to modeling survival with cancer therapies have been proposed, and the cure model was used to model the conditional survival function in this cost-effectiveness analysis. Cost-effectiveness analyses using a cure model approach are widely performed [26, 27]; such an approach has been used by the National Institute for Health and Care Excellence (NICE) for health technology assessments in the UK [28, 29]. The survival model explicitly incorporates the colon cancer cure rate and considers the risk of death by causes other than colon cancer. The estimated survival curve fits well with the nonparametric curve of the trial results. The estimated RFS and OS were similar to those reported in the JFMC37-0801 study; the estimated and reported 5-year RFS rates were 74.4% and 74.1% with the 12-month capecitabine regimen, and 69.8 and 69.3% with the 6-month regimen. Estimated and reported 5-year OS rates were 83.2% and 83.2% with the 6-month capecitabine regimen, and 88.1% and 87.6% with the 12-month regimen. The cure rates estimated from the JFMC37-0801 study were 0.726 and 0.694 with 12- and with 6-month regimens, respectively. These were validated by the recurrence rate of stage III CRC (31.8%) reported in the Japanese guidelines for CRC [2].

Although the utility of the 12-month capecitabine regimen was expected to be lower than that of the 6-month course due to the extension of treatment duration, it was similar in both groups. In the JFMC37-0801 study, the incidence of HFS was higher with 12-month capecitabine, but the incidence of other grade 3–4 AEs was comparable, and therefore, the utility of 12-month capecitabine might not have decreased during the treatment period. There was no decrease in QOL due to the extension of the capecitabine treatment period. Since the JFMC37-0801 only evaluated up to 5 years, the utility values for the general population were those determined after 5 years. This assumption was adopted for a conservative setting because the utility values estimated from the JFMC37-0801 study were higher than the QOL values of the general population.

As postoperative adjuvant chemotherapy for patients with stage III CRC, CAPOX (capecitabine plus oxaliplatin) is widely accepted as the international standard postoperative adjuvant chemotherapy for stage III CRC [4–6]. On the other hand, oxaliplatin has been rejected by some patients due to its associated neurotoxicity. Recently, a shortened course (3 months) of oxaliplatin was investigated to avoid peripheral neuropathy, and promising results were obtained [30]. It has been reported that the shortened oxaliplatin regimen is expected to reduce QALY losses due to AEs and reduce treatment costs due to the shorter treatment period. On the other hand, oral fluoropyrimidine monotherapy is preferred for adjuvant therapy in Japan, and Japanese RCTs have shown favorable results [7–10]. In these studies, 6 months of treatment has also been considered as standard, but the possibility that an extended treatment period of 12 months may improve prognosis has been shown [11], and the results of the study suggest that 12 months of capecitabine monotherapy may be an adjuvant treatment option, especially for patients who are prone and averse to neurotoxicity. Although the results of this analysis show additional drug costs due to the extended duration of capecitabine treatment, the lifetime total cost of the 12-month regimen was cost-saving and could be a treatment option if clinical benefits are demonstrated for the 12-month capecitabine course.

There are some limitations of the prospectively collected RCT data. First, the utility score of the post-metastasis period was not captured directly from patients who experienced recurrence; it was based on published reports. Second, the post-metastasis costs were calculated from the data of only 25 cases, and because the partitioned survival model was used, we could not apply a discount rate to the recurrence costs. However, since most recurrences occur early, up to 5 years after the start of the analysis, the impact of not considering the discount rate is considered to be limited. In addition, the sensitivity analysis suggested that these limitations had little impact on the results.

Another major limitation was that the JFMC37-0801 study did not compare between 12 months of capecitabine and the oxaliplatin-containing regimen, a current global standard for adjuvant treatment of stage III colon cancer. The JFMC37-0801 study demonstrated significantly better OS and RFS associated with the 12-month capecitabine regimen compared with the standard 6-month treatment, and the obtained OS and RFS were comparable to those in the pivotal study of the 6-month oxaliplatin regimens [4–6]. Our JFMC37-0801 auxiliary study found no QOL-related concerns associated with the prolonged treatment period, and the 12-month capecitabine course was more cost-effective than the 6-month course, considering drug costs, the costs of treating AEs, and the costs of treating relapses. In recent years, a shortened (3-month) oxaliplatin regimen (aimed at avoiding peripheral neuropathy) was investigated, and in patients treated with CAPOX, 3 months of therapy was as effective as 6 months, particularly in the lower-risk subgroup, and a shorter duration of adjuvant treatment was associated with a significantly lower incidence of AEs than a longer duration [30]. Twelve months of capecitabine therapy is a favorable option for improving prognosis and avoiding oxaliplatin-associated toxicity. Since no studies have compared these approaches (to avoiding oxaliplatin toxicity), future studies evaluating these treatment strategies are needed.

## Conclusions

Based on the cost-effectiveness analysis for patients with stage III colon cancer from the Japanese public healthcare payer’s perspective, the 12-month capecitabine regimen was found dominant to the 6-month regimen. Twelve months of capecitabine was the favorable option of postoperative adjuvant chemotherapy for stage III colon cancer from the perspective of cost-effectiveness.

## Supplementary Information

Below is the link to the electronic supplementary material.

Supplementary file1 (DOC 633 KB)

## Data Availability

The data that support the findings of this study are available from the corresponding author upon reasonable request.

## Code availability

Code sharing not applicable to this article.
