# Supplementary material for: Cost-effectiveness of 12 months of capecitabine as adjuvant chemotherapy for stage III colon cancer: preplanned cost-effectiveness analysis of the JFMC37-0801 study
Source: Eur J Health Econ. 2022 Jan 24;23(7):1159–71. doi: 10.1007/s10198-021-01418-6 (PMC9395498; doi:10.1007/s10198-021-01418-6)
Supplement: Supplementary file 1 — Supplementary file1 (DOC 633 KB) [file 10198_2021_1418_MOESM1_ESM.doc]

Supplementary Table 1. Patient demographics

|  |  | JFMC37-0801 (intension-to-treat) | | | | | | HRQOL population | | | | | |  | | Cost population | | | | | | |
| --- | --- | --- | --- | --- | --- | --- | --- | --- | --- | --- | --- | --- | --- | --- | --- | --- | --- | --- | --- | --- | --- | --- |
|  |  | 12 months | |  | 6 months | | | 12 months | |  | | 6 months | |  | | 12 months | | |  | | 6 months | |
|  |  | n = 650 | |  | n = 654 | | | n = 90 | |  | | n = 81 | |  | | n = 61 | | |  | | n = 47 | |
| Nodal status | N1 | 498 | (76.6) |  | 504 | (77.1) |  | 66 | (73.3) |  | 59 | | (72.8) | |  | | 45 | (73.8) | |  | 35 | (74.5) |
|  | N2/N3 | 152 | (23.4) |  | 150 | (22.9) |  | 24 | (26.7) |  | 22 | | (27.2) | |  | | 16 | (26.2) | |  | 12 | (25.5) |
| Sex | Male | 343 | (52.8) |  | 352 | (53.8) |  | 44 | (48.9) |  | 44 | | (54.3) | |  | | 28 | (45.9) | |  | 29 | (61.7) |
|  | Female | 307 | (47.2) |  | 302 | (46.2) |  | 46 | (51.1) |  | 37 | | (45.7) | |  | | 33 | (54.1) | |  | 18 | (38.3) |
| Age | <70 | 442 | (68.0) |  | 451 | (69.0) |  | 59 | (65.6) |  | 60 | | (74.1) | |  | | 37 | (60.7) | |  | 31 | (66) |
|  | ≧70 | 208 | (32.0) |  | 203 | (31.0) |  | 31 | (34.4) |  | 21 | | (25.9) | |  | | 24 | (39.3) | |  | 16 | (34) |
| Tumor location | Right-sided | 263 | (40.5) |  | 262 | (40.1) |  | 40 | (44.4) |  | 34 | | (42) | |  | | 28 | (45.9) | |  | 20 | (42.6) |
|  | Left-sided | 252 | (38.8) |  | 258 | (39.4) |  | 36 | (40) |  | 33 | | (40.7) | |  | | 19 | (31.2) | |  | 18 | (38.3) |
|  | Rectosigmoid | 135 | (20.8) |  | 134 | (20.5) |  | 14 | (15.6) |  | 14 | | (17.3) | |  | | 14 | (23) | |  | 9 | (19.2) |
| Surgical approach | Laparoscopic | 255 | (39.2) |  | 276 | (42.2) |  | 41 | (45.6) |  | 39 | | (48.2) | |  | | 29 | (47.5) | |  | 18 | (38.3) |
|  | Open (conventional) | 395 | (60.8) |  | 378 | (57.8) |  | 49 | (54.4) |  | 42 | | (51.9) | |  | | 32 | (52.5) | |  | 29 | (61.7) |
| Histological type | Pap/Well | 178 | (27.4) |  | 189 | (28.9) |  | 24 | (26.7) |  | 25 | | (30.9) | |  | | 16 | (26.2) | |  | 18 | (38.3) |
|  | Tub/Mod | 433 | (66.6) |  | 419 | (64.1) |  | 60 | (66.7) |  | 46 | | (56.8) | |  | | 40 | (65.6) | |  | 25 | (53.2) |
|  | Other | 39 | (6.0) |  | 46 | (7.0) |  | 6 | (6.7) |  | 10 | | (12.4) | |  | | 5 | (8.2) | |  | 4 | (8.5) |
| T (TNM 7th) | T1/T2 | 100 | (15.4) |  | 101 | (15.4) |  | 11 | (12.2) |  | 13 | | (16.1) | |  | | 8 | (13.1) | |  | 6 | (12.8) |
|  | T3 | 363 | (55.8) |  | 366 | (56.0) |  | 37 | (41.1) |  | 40 | | (49.4) | |  | | 26 | (42.6) | |  | 26 | (55.3) |
|  | T4 | 187 | (28.8) |  | 187 | (28.6) |  | 42 | (46.7) |  | 28 | | (34.6) | |  | | 27 | (44.3) | |  | 15 | (31.9) |
| N (TNM 7th) | N1 | 506 | (77.8) |  | 512 | (78.3) |  | 67 | (74.4) |  | 59 | | (72.8) | |  | | 45 | (73.8) | |  | 35 | (74.5) |
|  | N2 | 144 | (22.2) |  | 142 | (21.7) |  | 23 | (25.6) |  | 22 | | (27.2) | |  | | 16 | (26.2) | |  | 12 | (25.5) |
| Stage (TNM 7th) | IIIA | 96 | (14.8) |  | 94 | (14.4) |  | 10 | (11.1) |  | 12 | | (14.8) | |  | | 8 | (13.1) | |  | 6 | (12.8) |
|  | IIIB | 458 | (70.5) |  | 461 | (70.5) |  | 60 | (66.7) |  | 53 | | (65.4) | |  | | 37 | (60.7) | |  | 30 | (63.8) |
|  | IIIC/IV | 96 | (14.8) |  | 99 | (15.1) |  | 21 | (23.3) |  | 16 | | (19.8) | |  | | 17 | (27.9) | |  | 11 | (23.4) |

Supplementary Table 2. Number of patients and collection rate at each time point

|  | Survival analysis | | |  | QOL analysis | | | | |  | Cost analysis | | | | |
| --- | --- | --- | --- | --- | --- | --- | --- | --- | --- | --- | --- | --- | --- | --- | --- |
|  | 12 months |  | 6 months |  | 12 months | |  | 6 months | |  | 12 months | |  | 6 months | |
| month 1 | 649 |  | 654 |  | 87/90 | (96.7) |  | 79/81 | (97.5) |  | 60/61 | (98.4) |  | 46/47 | (97.9) |
| month 3 | 649 |  | 651 |  | 83/90 | (92.2) |  | 76/81 | (93.8) |  | 59/61 | (96.7) |  | 45/47 | (95.7) |
| month 6 | 648 |  | 648 |  | 76/90 | (84.4) |  | 69/80 | (86.3) |  | 57/61 | (93.4) |  | 44/47 | (93.6) |
| month 9 | 648 |  | 647 |  | 71/89 | (79.8) |  | 61/73 | (83.6) |  | 50/60 | (83.3) |  | 25/47 | (53.2) |
| month 12 | 642 |  | 647 |  | 70/87 | (80.5) |  | 56/67 | (83.6) |  | 51/60 | (85.0) |  | 27/47 | (57.4) |
| month 15 | 638 |  | 641 |  | 66/86 | (76.7) |  | 48/64 | (75.0) |  | 34/60 | (56.7) |  | 26/46 | (56.5) |
| month 18 | 637 |  | 637 |  | 58/82 | (70.7) |  | 47/64 | (73.4) |  | 31/60 | (51.7) |  | 22/46 | (47.8) |
| month 24 | 625 |  | 621 |  | 64/81 | (79.0) |  | 49/61 | (80.3) |  | 38/59 | (64.4) |  | 20/45 | (44.4) |
| month 36 | 599 |  | 586 |  | 52/78 | (66.7) |  | 41/57 | (71.9) |  | 35/59 | (59.3) |  | 17/42 | (40.5) |
| month 48 | 576 |  | 551 |  | 50/75 | (66.7) |  | 35/54 | (64.8) |  | 24/58 | (41.4) |  | 12/40 | (30.0) |
| month 60 | 540 |  | 515 |  | 48/75 | (64.0) |  | 38/53 | (71.7) |  | 17/55 | (30.9) |  | 10/36 | (27.8) |

Supplementary Table 3. Monthly cost for metastasis

|  | N | Cost (JPY) |  |  | N | Cost (JPY) |
| --- | --- | --- | --- | --- | --- | --- |
| month 1 | 25 | 581,104 |  | month 25 | 17 | 189,802 |
| month 2 | 24 | 491,112 |  | month 26 | 16 | 251,136 |
| month 3 | 24 | 348,639 |  | month 27 | 16 | 225,975 |
| month 4 | 24 | 324,907 |  | month 28 | 16 | 210,512 |
| month 5 | 24 | 440,650 |  | month 29 | 16 | 176,304 |
| month 6 | 24 | 255,110 |  | month 30 | 16 | 264,955 |
| month 7 | 24 | 272,827 |  | month 31 | 15 | 182,221 |
| month 8 | 24 | 221,791 |  | month 32 | 13 | 191,101 |
| month 9 | 24 | 242,507 |  | month 33 | 12 | 168,173 |
| month 10 | 24 | 199,675 |  | month 34 | 12 | 137,214 |
| month 11 | 23 | 126,273 |  | month 35 | 12 | 72,175 |
| month 12 | 23 | 146,708 |  | month 36 | 12 | 110,153 |
| month 13 | 23 | 137,661 |  | month 37 | 12 | 130,657 |
| month 14 | 22 | 177,289 |  | month 38 | 11 | 94,144 |
| month 15 | 20 | 236,508 |  | month 39 | 10 | 122,733 |
| month 16 | 20 | 248,377 |  | month 40 | 9 | 79,780 |
| month 17 | 20 | 292,138 |  | month 41 | 9 | 40,886 |
| month 18 | 20 | 244,144 |  | month 42 | 9 | 80,605 |
| month 19 | 20 | 154,527 |  | month 43 | 9 | 74,018 |
| month 20 | 18 | 241,888 |  | month 44 | 8 | 88,599 |
| month 21 | 18 | 119,625 |  | month 45 | 7 | 177,738 |
| month 22 | 18 | 214,881 |  | month 46 | 6 | 198,995 |
| month 23 | 18 | 183,063 |  | month 47 | 6 | 59,606 |
| month 24 | 18 | 185,674 |  | month 48 | 5 | 68,533 |

As the number of patients was less than five after 48 months, the monthly cost of relapse for months 48 to 60 were assumed to be the same as that of month 48.

Supplementary Figure 1. CONSORT diagram of QOL and cost population


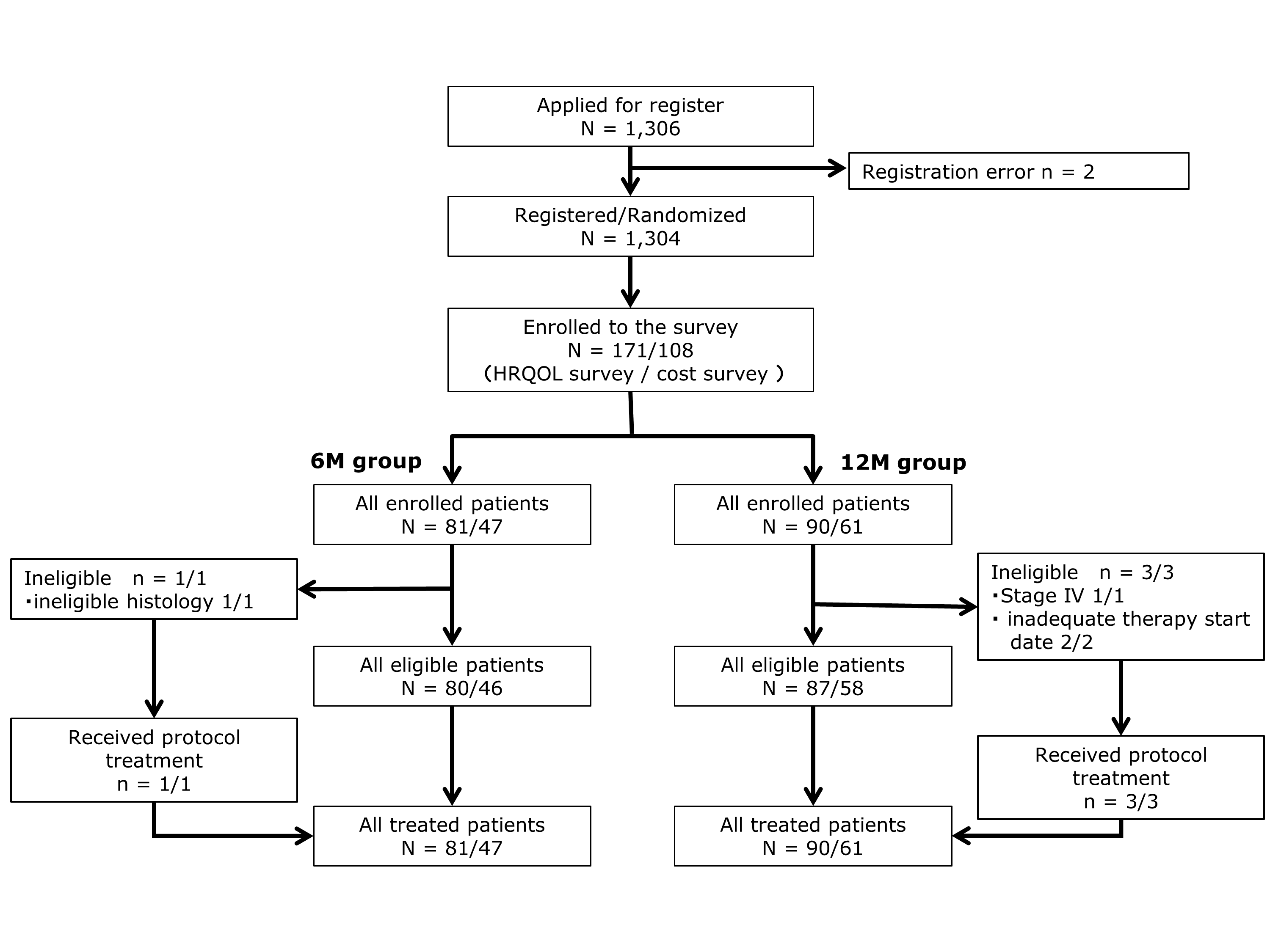


Supplementary Figure2. Survival curve of recurrent patients


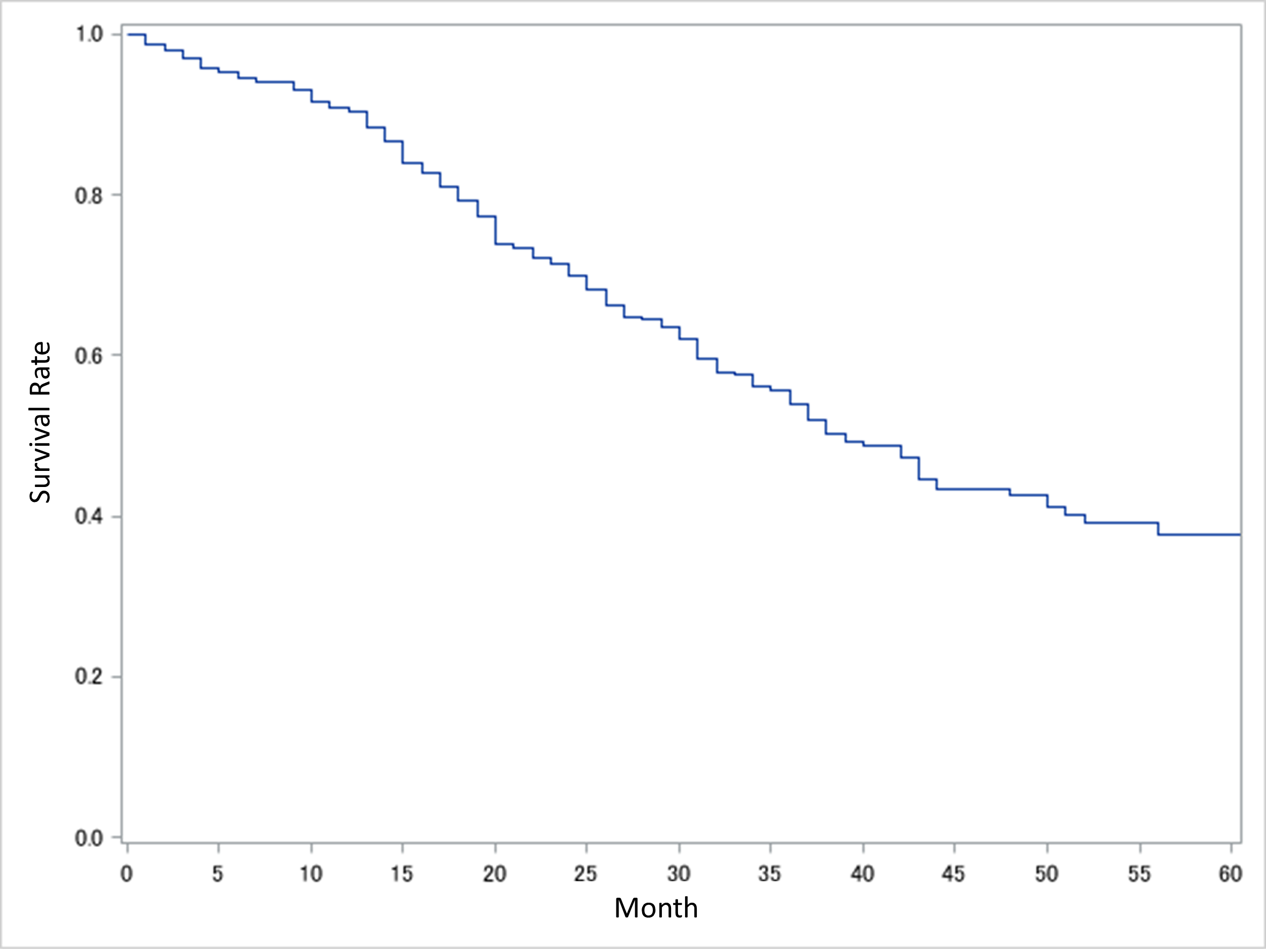


Supplementary Figure3. Standard parametric OS curves


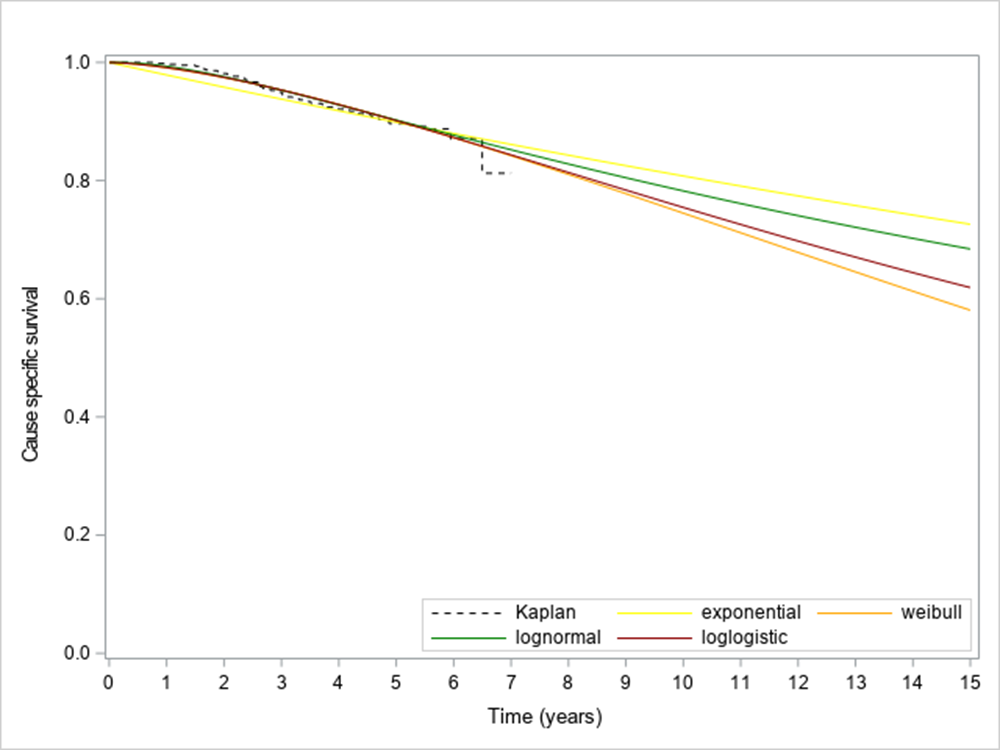


(a) 12 months Capecitabine


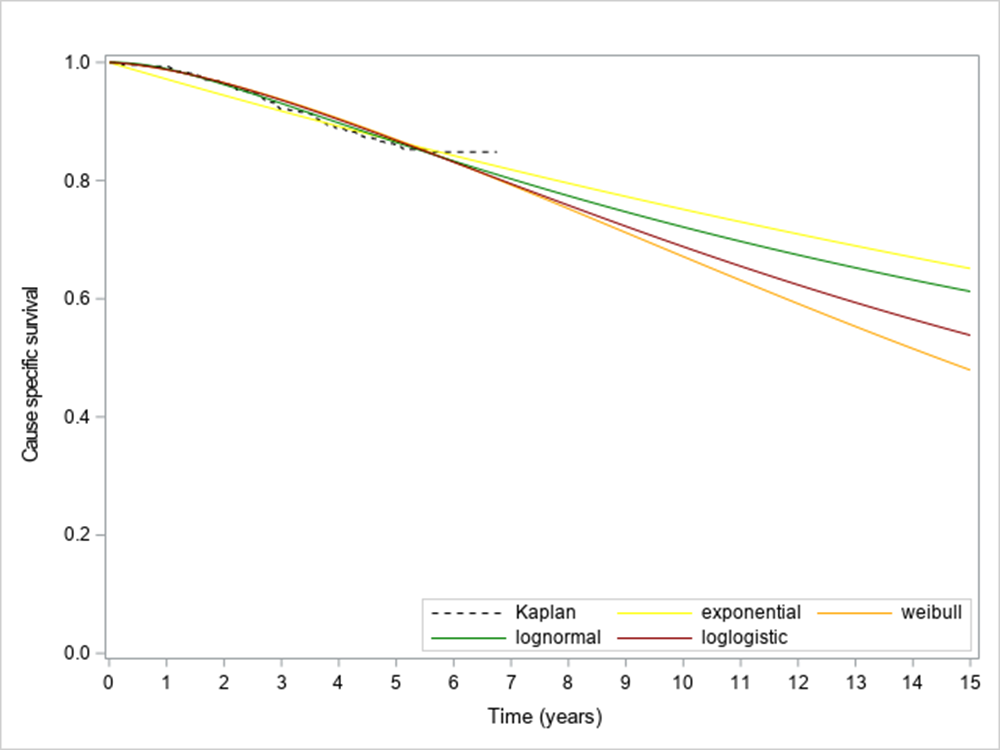


(b) 6 months Capecitabine
